# Supplementary material for: Dimerization processes for light-regulated transcription factor Photozipper visualized by high-speed atomic force microscopy
Source: Sci Rep. 2022 Aug 8;12:12903. doi: 10.1038/s41598-022-17228-6 (PMC9359980; doi:10.1038/s41598-022-17228-6)
Supplement: Supplementary file 1 — Supplementary Information 1. [file 41598_2022_17228_MOESM1_ESM.pdf]

Description of Additional Supplementary Files

Supplementary Movies

Supplementary Movie 1

HS-AFM movie of PZ molecules on the mica surface under dark conditions. 100×100 nm^2, 200×200 pixels, 1.0 sec/frame, played ×2.

Supplementary Movie 2

HS-AFM movie of PZ molecules on the mica surface under continuous light illumination. 100×100 nm^2, 200×200 pixels, 0.5 sec/frame, played ×2.

Supplementary Movie 3

HS-AFM movie of PZ-S_2C molecules on the mica surface under continuous light illumination. 100×100 nm^2, 200×200 pixels, 0.5 sec/frame, played ×2.

Supplementary Movie 4

HS-AFM movie of a PZ dimer molecule on the mica surface under continuous light illumination. Two LOV domain monomers are indicated with white arrowheads. Filmstrips for this movie are shown in Supplementary Figure 4. 22.5×22.5 nm^2, 45×45 pixels, 0.5 sec/frame, played ×1.

Supplementary Movie 5

HS-AFM movie of PZ molecules on the mica surface under dark conditions. This movie was used for the analyses of representative trajectories in Fig. 3a. 150×150 nm^2, 200×200 pixels, 0.5 sec/frame, played ×5.

Supplementary Movie 6

HS-AFM movie of PZ molecules on the mica surface under light conditions. This movie was used for the analyses of representative trajectories in Fig. 3b. 150×150 nm^2, 200×200 pixels, 0.5 sec/frame, played ×5.

Supplementary Movie 7

HS-AFM movie of PZ molecules transitioning from dark to light state. Time displays in the AFM movie indicate time course after blue light illumination. 150×150 nm^2, 200×200 pixels, 0.5 sec/frame. The playback speed is indicated in movie.

Supplementary Movie 8

HS-AFM movie of the dimerization process of PZ molecules observed under continuous light illumination. 60×60 nm^2, 80×80 pixels, 0.5 sec/frame, played ×1.0.

Supplementary Movie 9

HS-AFM movie of the dissociation process of a PZ dimer molecule observed under continuous light illumination. 52.5×52.5 nm^2, 70×70 pixels, 0.5 sec/frame, played ×1.0.
