# Supplementary material for: Dimerization processes for light-regulated transcription factor Photozipper visualized by high-speed atomic force microscopy
Source: Sci Rep. 2022 Aug 8;12:12903. doi: 10.1038/s41598-022-17228-6 (PMC9359980; doi:10.1038/s41598-022-17228-6)
Supplement: Supplementary file 2 — Supplementary Information 2. [file 41598_2022_17228_MOESM2_ESM.pdf]

Supplementary Information

Dimerization processes for light-regulated transcription factor Photozipper visualized by high-speed atomic force microscopy

Akihiro Tsuji, Hayato Yamashita,
Osamu Hisatomi, Masayuki Abe

Supplementary Figure 1

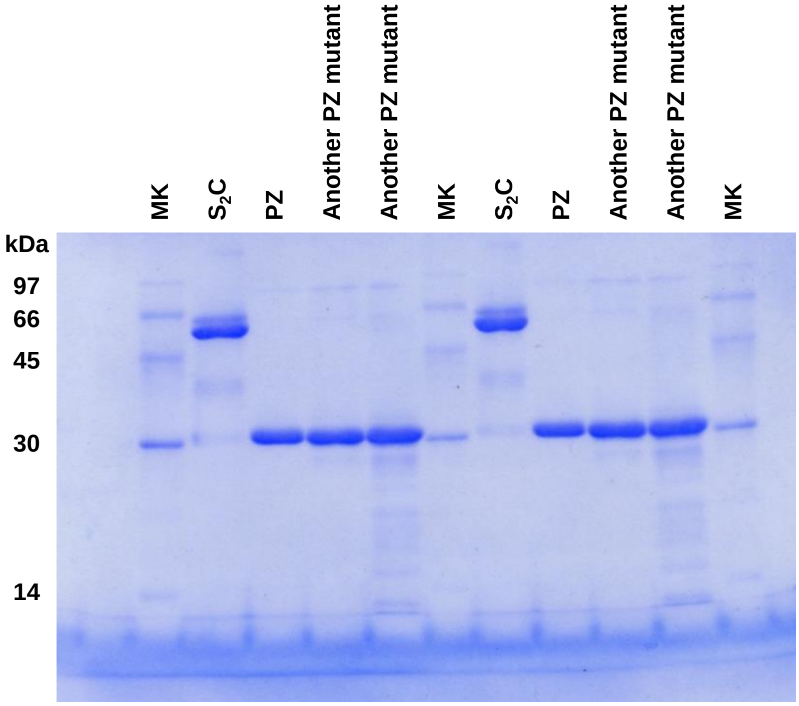

Supplementary Figure 1. Original image of the full-length membrane used for the SDS-PAGE. This picture is the original image of SDS-PAGE shown in Fig. 1c. The lanes for the marker(MK), S_2C and PZ are included as indicated. This SDS-PAGE were performed under non-reducing conditions.

Supplementary Figure 2

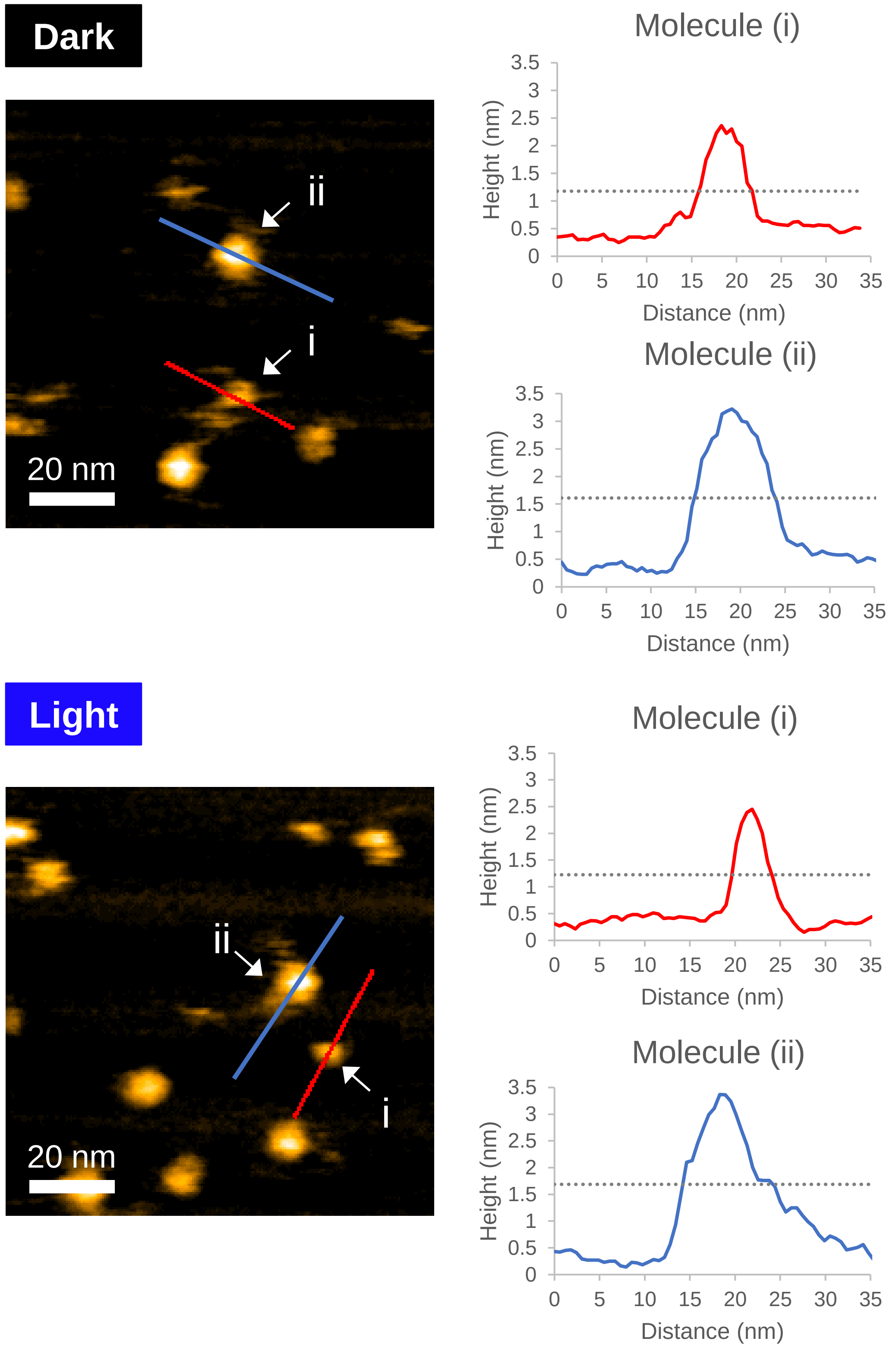

Supplementary Figure 2. AFM image of wild-type PZ molecules observed under dark and light conditions and cross-sectional analyses.
The cross-sections of two molecules in AFM images are shown under dark and light conditions, respectively. Dotted line in each graph indicates the half-maximum height for each profile. Half maximum full-width: Dark, (Molecule (i)) 5.7 nm, (Molecule (ii)) 9.5 nm. Light, (Molecule (i)) 4.6 nm, (Molecule (ii)) 10.4 nm. Scan rates: Dark, 1.0 sec/frame. Light, 0.5 sec/frame.

Supplementary Figure 3

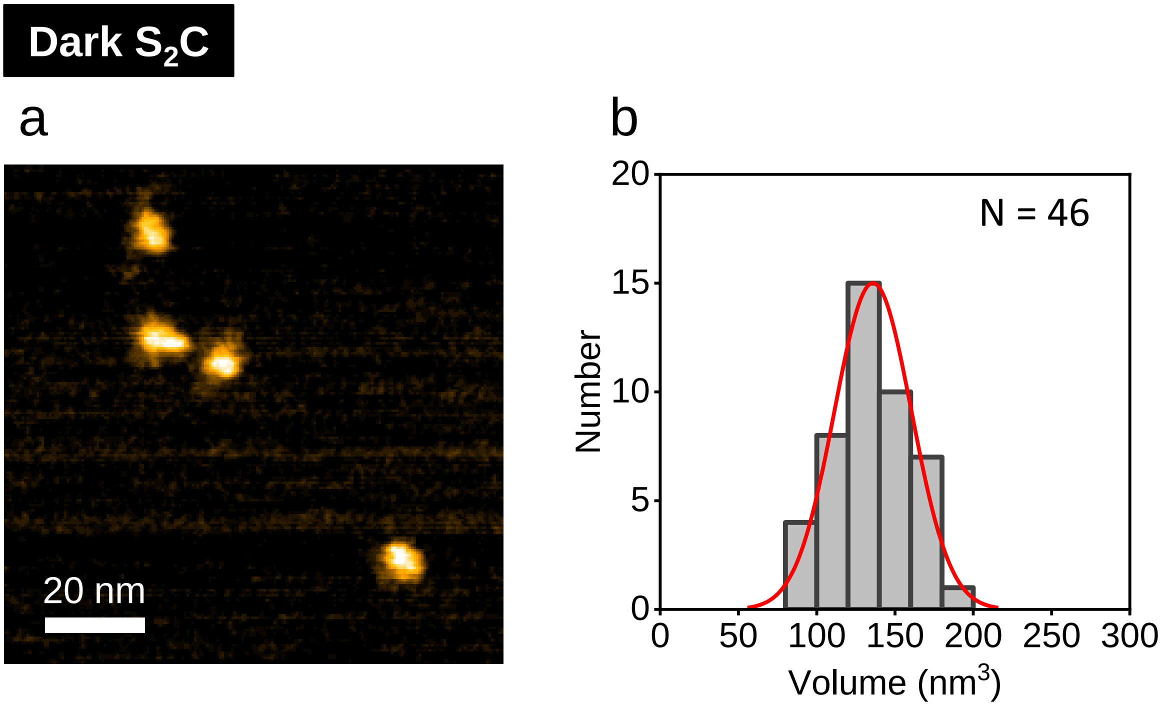

Supplementary Figure 3. HS-AFM observation of S_2C under dark.
(a) HS-AFM images of S_2C molecules observed under dark conditions. (b) Histogram for the molecular volumes analyzed from AFM images of S_2C under dark conditions. N indicates the number analyzed for different molecules in AFM images. Curve represents the fit to a Gaussian distribution. Mean value of the Gaussian curve: 136 nm^3. Protein concentration: 20 nM. Scan range: 100×100 nm^2 Scan rates: 0.5 sec/frame.

Supplementary Figure 4

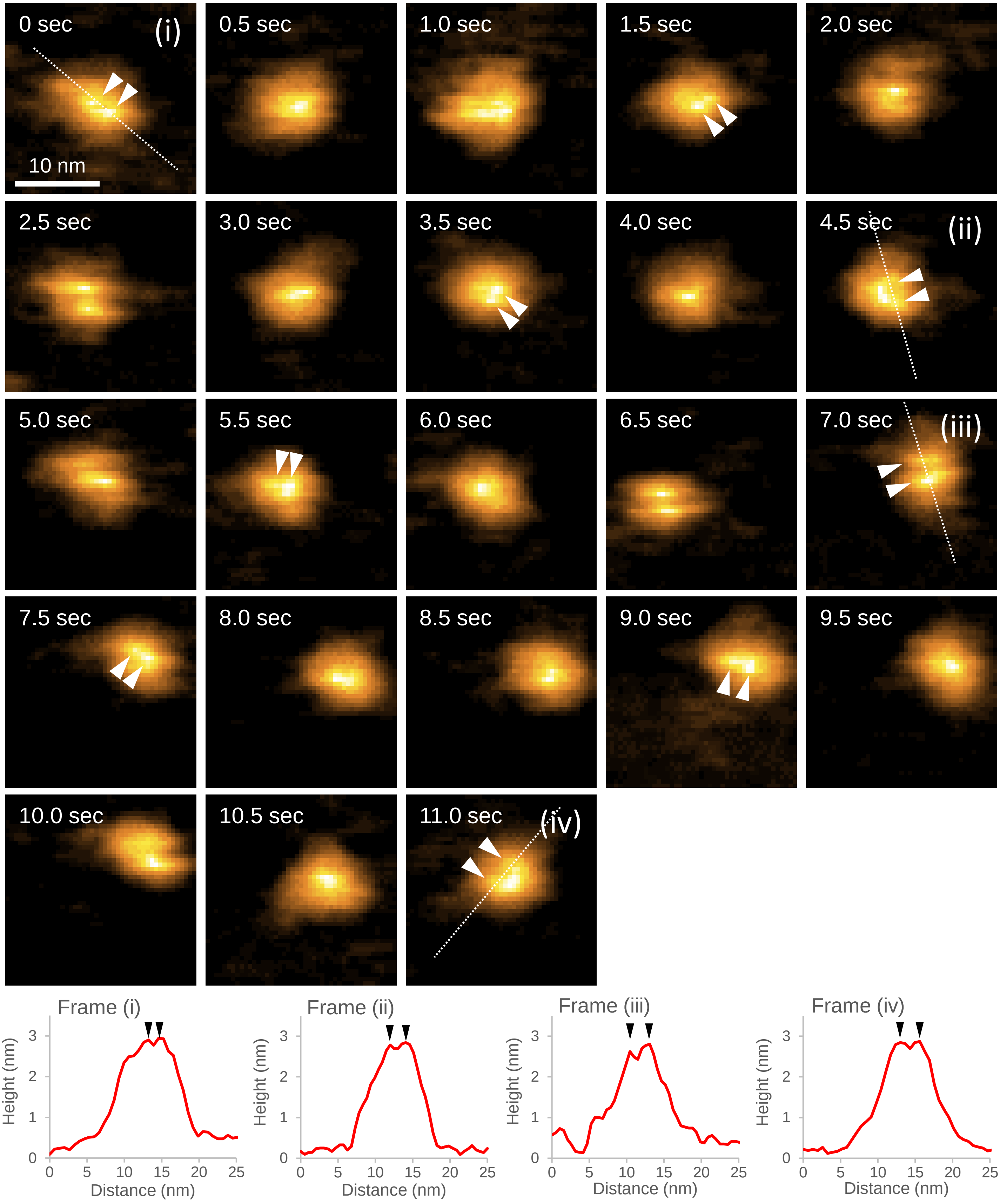

Supplementary Figure 4. Filmstrips of a successive AFM movie and cross-sectional analysis of a PZ dimer under continuous light illumination.

Two LOV domains are apparent within the PZ dimer as indicated by white arrowheads. Each cross-sectional analysis corresponds to the frame labeled with the same roman numeral. All the cross-sections exhibit two peaks (black arrowheads), supporting the appearance of two LOV domains. Scan rate: 0.5 sec/frame. See also Supplementary Movie 4.

Supplementary Figure 5

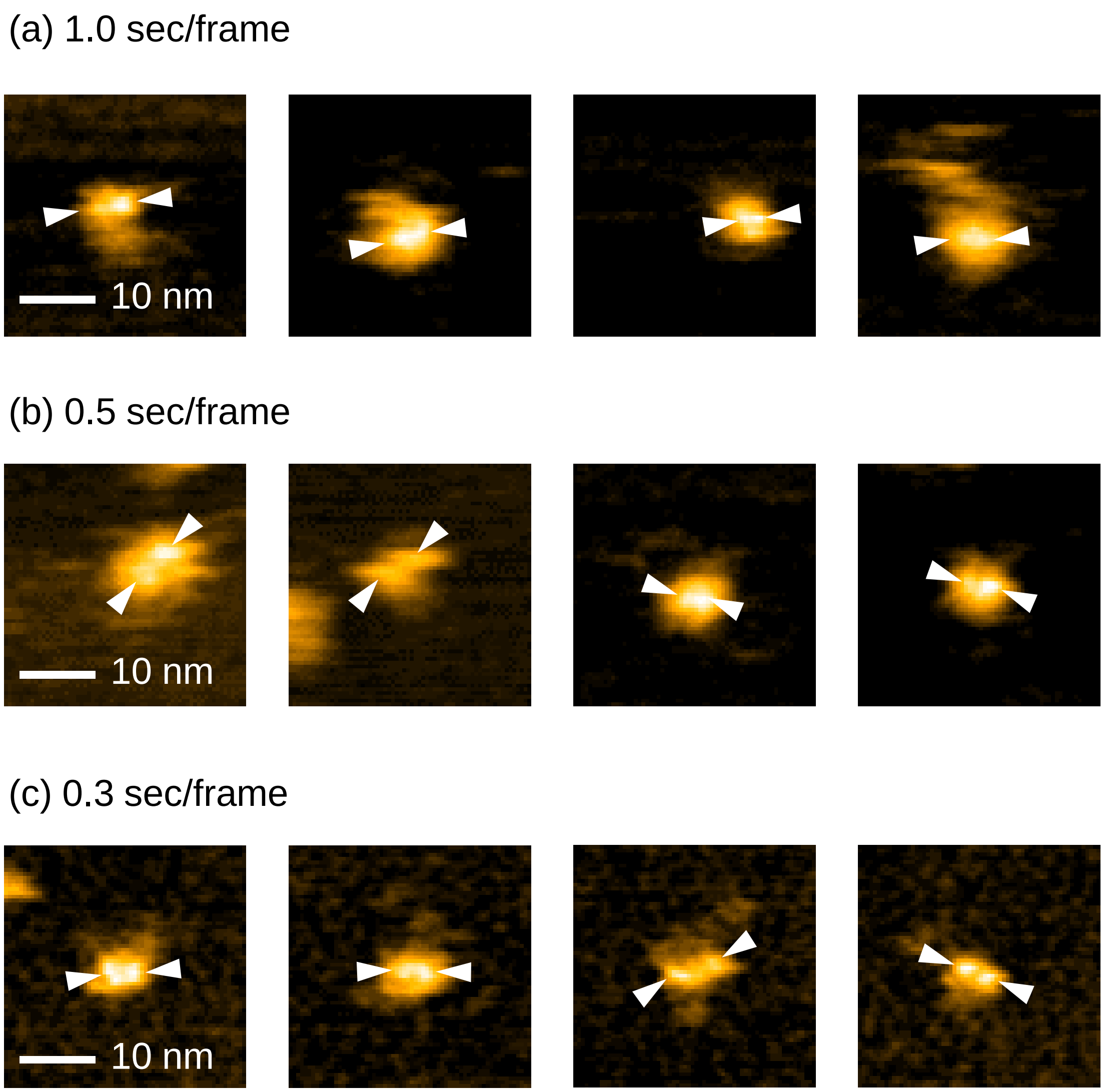

Supplementary Figure 5. HS-AFM images of bilobed PZ dimers under continuous light illumination.

Bilobed dimers observed at scan rates of (a) 1.0 sec/frame, (b) 0.5 sec/frame, (c) 0.3 sec/frame. Two LOV domains are apparent within the PZ dimer as indicated by white arrowheads. Such appearance of the LOV domains were observed at various scan rates among different molecules, also oriented in various directions.

Supplementary Figure 6

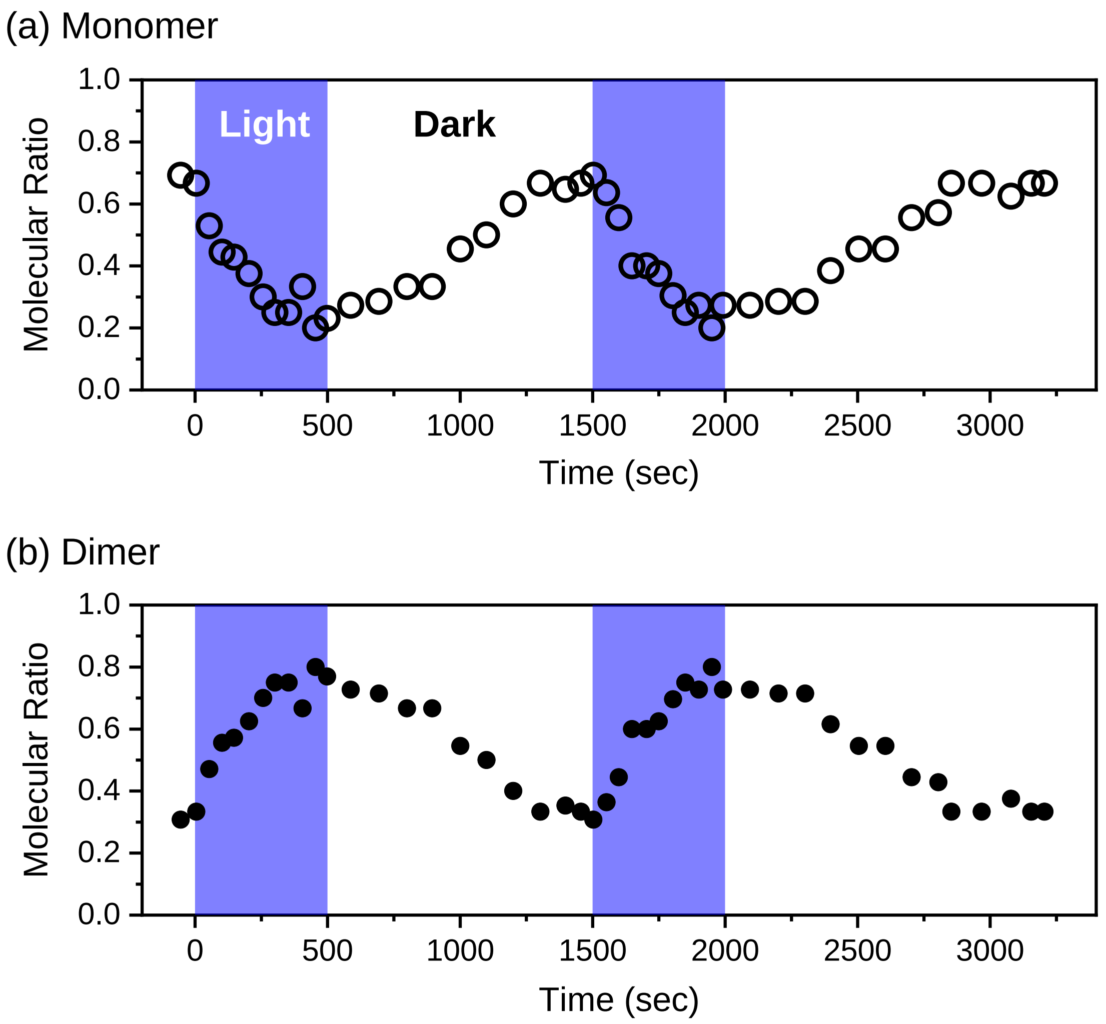

Supplementary Figure 6. Time course of each ratio of PZ monomers and dimers in light–dark repeated cycle.

Open and filled circles indicate the ratio of (a) monomers and (b) dimers, respectively, analyzed in a successive AFM movie. Each ratio was normalized by the total PZ molecules in each AFM image. Note that one PZ dimer was counted as two PZ molecules. The blue regions show periods of blue light (BL) illumination (450 nm). Under dark conditions before initial BL illumination (< 0 sec), the monomer ratio was predominant. After initial BL illumination, the dimer ratio gradually increased with time, while the monomer ratio decreased with time. Under dark conditions after finishing the initial BL illumination, the dimer ratio gradually decreased with time, while the monomer ratio increased with time. At ~1300 sec, each PZ ratio returned to the initial state. Similar responses were reproduced in second light-dark cycle. These HS-AFM measurements were performed in a solution (Buffer-C) containing 200 mM NaCl, 20 mM Tris-HCl (pH 7), 1 mM DTT, 0.2 mM PMSF. Buffer-C, having ionic strength close to intracellular conditions, was used in this experiment to observe repeatedly the physiological process of PZ as reversible monomer-dimer transitions on an AFM stage.

Supplementary Figure 7

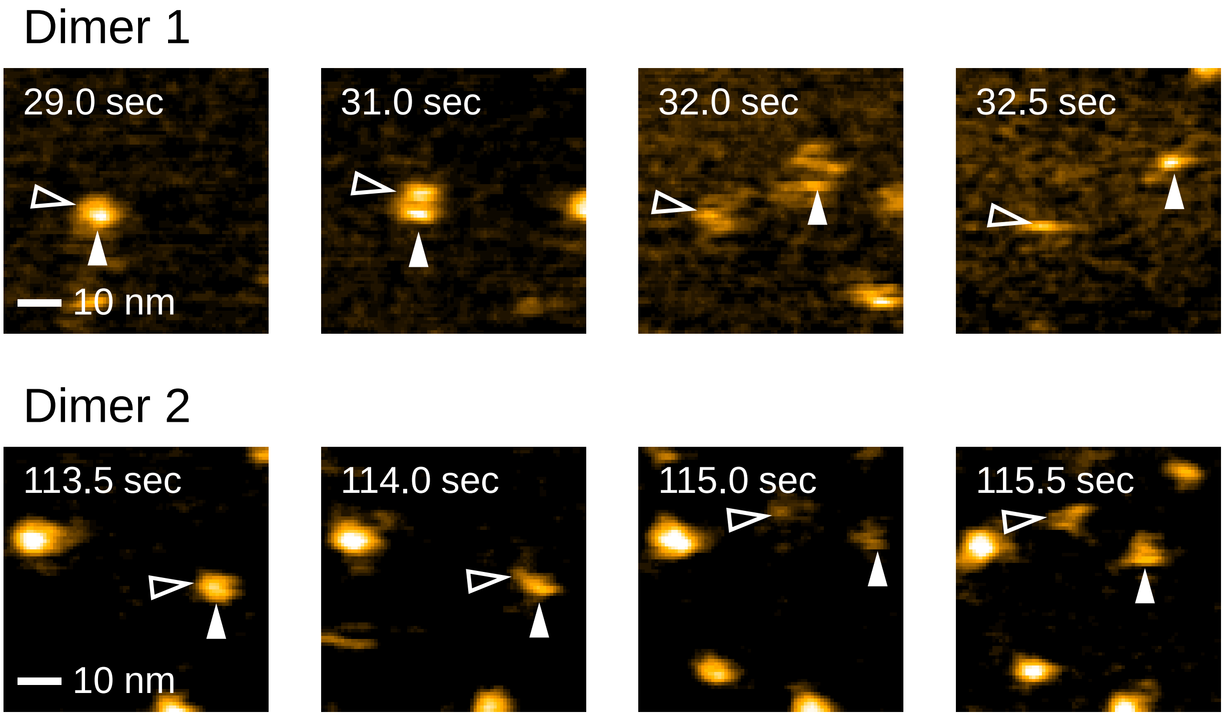

Supplementary Figure 7. Snapshots of successive AFM movies captured the dissociation processes of PZ dimer under BL illumination.

Dimers 1 and 2 dissociated into monomers at 32.0 and 115.0 sec, respectively. Each open and filled arrowheads indicate individual PZ monomers. See also Supplementary Movie 9 for Dimer 1. Scan rates: 0.5 sec/frame.

Supplementary Figure 8

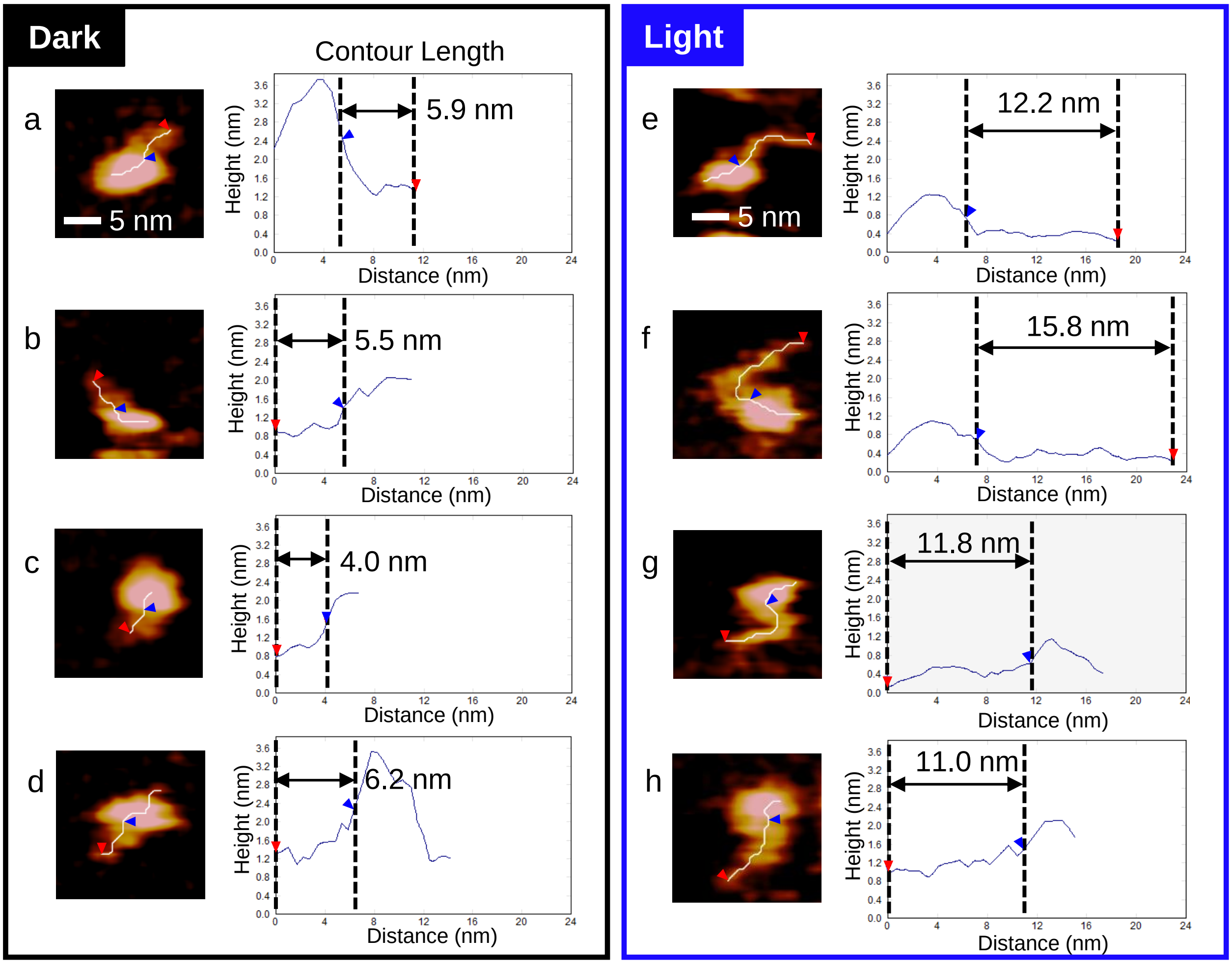

Supplementary Figure 8. Analysis of the contour lengths in PZ monomers.
(a–d) HS-AFM images of PZ monomers under dark conditions. These images correspond to that of Fig. 5(b). (e)–(h) HS-AFM images of PZ monomers under light conditions. These images correspond to that of Fig. 5(e). The skeleton lines are drawn in white on each PZ monomer. The procedure is described in Methods. Line profiles along the skeleton lines are shown next to each AFM image. The half-maximum points (positions at blue arrowheads) were used as a threshold to separate LOV domain from bZIP-linker region in each profile. Red arrowheads indicate the bZIP termini. Lengths of the white line between the blue and red arrowheads were considered as bZIP-linker contour lengths.

Supplementary Note –Appendix

The overall reaction of PZ forming the monomer and dimer under dark and light conditions can be explained by the model as shown in Supplementary Fig. 9. Under steady state conditions of dark, PZ monomers and dimers of dark state are in equilibrium. After blue light (BL) illumination, PZ molecules transition to dark state monomers and dimers. Consequently, the equilibrium of PZ shifts from the dark state to the light state (from the gray area to the blue area in Supplementary Fig. 9). This reaction is reversible. Therefore, after turning off the lights, the equilibrium of PZ returns to the initial states. Since PZ can be activated by BL within a matter of milliseconds, most of the monomers and dimers are likely to be quickly in the light state after continuous BL illumination[1]. Thus, we considered that the transient change from dark to light can be simplified into an equilibrium reaction between the light state monomer and the light state dimer.

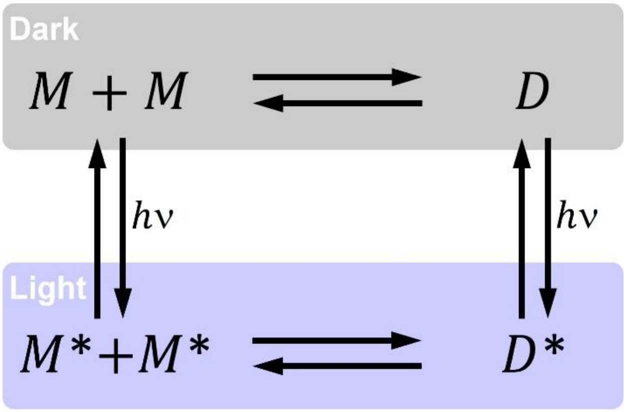

Supplementary Figure 9: Photo-induced transition model of PZ between dark and light. $\boldsymbol{M}$: dark state monomer, $\boldsymbol{M}*$: light-state monomer, $\boldsymbol{D}$: dark state dimer, $\boldsymbol{D}*$: light state dimer. Arrows with $h\nu$ indicate BL-activation. PZ monomers and dimers are in equilibrium under steady state conditions of dark and light, respectively.

The rate constant of the dimerization and monomerization of this equilibrium are each represented as k_+ and k_-, respectively (Equation (1)).

$$\mathbf{M}* + \mathbf{M}* \underset{k_-}{\overset{k_+}{\rightleftarrows}} \mathbf{D}* \tag{1}$$

We considered the rate equation of the light state dimer under continuous BL illumination to be as

$$\frac{dD*}{dt} = k_+ M*^2 - k_- D* \tag{2}$$

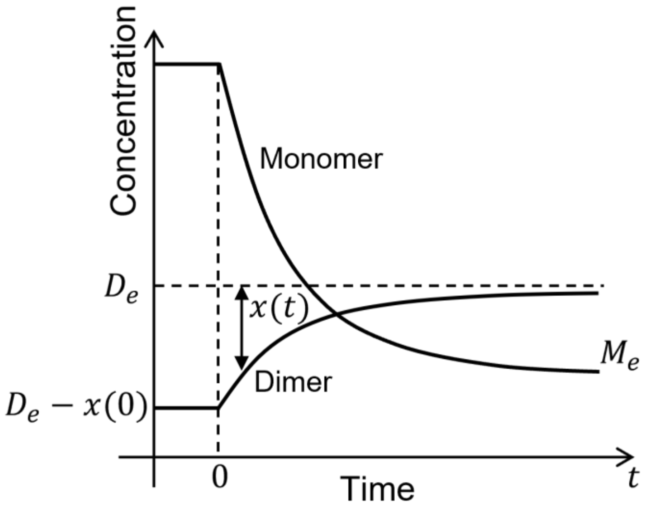

Supplementary Figure 10: Schematic diagram of the PZ monomer and dimer concentration development against time.

We rewrite $M*$ and $D*$ in equation (2) in terms of the difference from the equilibrium value (Supplementary Fig. 10):

$$D* = D_e - x(t) \tag{3}$$

$$M* = M_e + 2x(t) \tag{4}$$

M_e and D_e are monomer and dimer concentration at equilibrium, respectively (Supplementary Fig. 10). We then substitute $M*$ and $D*$ in equation (2) using equations (3) and (4).

$$\frac{d(D_e - x)}{dt} = k_+(M_e + 2x)^2 - k_-(D_e + x)$$

$$-\frac{dx}{dt} = k_+(M_e^2 + 4M_e x + 4x^2) - k_- D_e + k_- x \tag{5}$$

Due to the fact that the monomerization and dimerization rates are equal at equilibrium,

$$\frac{dD*}{dt} = k_+ {M_e}^2 - k_- D_e = 0 \tag{6}$$

We then apply equation (6) to (5),

$$-\frac{dx}{dt} = k_+(4M_e x + 4x^2) + k_- x$$

$$-\frac{dx}{dt} = 4k_+ x^2 + 4k_+ M_e x + k_- x$$

$$\frac{dx}{dt} = -4k_+ x(x + M_e + \frac{k_-}{4k_+})$$

For $x \neq 0$, we separate x and t,

$$\frac{1}{x\left(x + M_e + \frac{k_-}{4k_+}\right)} dx = -4k_+ dt$$

$$\int_{x(0)}^{x} \frac{1}{x\left(x + M_e + \frac{k_-}{4k_+}\right)} dx = \int_0^t (-4k_+) dt$$

$$\int_{x(0)}^{x} \frac{1}{M_e + \frac{k_-}{4k_+}} \left(\frac{1}{x} - \frac{1}{x + M_e + \frac{k_-}{4k_+}} \right) dx = \int_0^t (-4k_+) dt$$

$$\int_{x(0)}^{x} \left(\frac{1}{x} - \frac{1}{x + M_e + \frac{k_-}{4k_+}} \right) dx = \int_0^t (-4k_+ M_e - k_-) dt$$

$$\left[ln \left| x + M_e + \frac{k_-}{4k_+} \right| - ln|x| \right]_{x(0)}^{x} = (4k_+ M_e + k_-)t$$

For $x > 0$,

$$\left[ln \left(x + M_e + \frac{k_-}{4k_+} \right) - ln(x) \right]_{x(0)}^{x} = (4k_+ M_e + k_-)t$$

$$ln \left\{ \left(\frac{x + M_e + \frac{k_-}{4k_+}}{x} \right) \left(\frac{x(0)}{x(0) + M_e + \frac{k_-}{4k_+}} \right) \right\} = (4k_+ M_e + k_-)t$$

$$\left(\frac{x + M_e + \frac{k_-}{4k_+}}{x} \right) \left(\frac{x(0)}{x(0) + M_e + \frac{k_-}{4k_+}} \right) = exp[(4k_+ M_e + k_-)t]$$

$$\frac{x + M_e + \frac{k_-}{4k_+}}{x} = \frac{x(0) + M_e + \frac{k_-}{4k_+}}{x(0)} exp[(4k_+M_e + k_-)t]$$

$$\frac{M_e + \frac{k_-}{4k_+}}{x} = \frac{x(0) + M_e + \frac{k_-}{4k_+}}{x(0)} exp[(4k_+M_e + k_-)t] - 1$$

$$\boldsymbol{x = \frac{x(0)\left(M_e + \frac{k_-}{4k_+}\right)}{\left(x(0) + M_e + \frac{k_-}{4k_+}\right) exp[(4k_+M_e + k_-)t] - x(0)}} \tag{7}$$

We then introduced M_{tot}, which is the total monomer PZ concentration. M_{tot} satisfies:

$$\boldsymbol{M_{tot} = M * + 2D * = M_e + 2D_e} \tag{8}$$

We then rewrote equation (7) without using M_e but by using M_{tot}. By taking the square of the relaxation time $1/\tau' = 4k_+M_e + k_-$, we get:

$$\boldsymbol{\frac{1}{\tau'^2} = (4k_+M_e + k_-)^2 = 16k_+^2M_e^2 + 8k_+k_-M_e + k_-^2 = 8k_+(2k_+M_e^2 + k_-M_e) + k_-^2} \tag{9}$$

From the equilibrium condition in equation (6),

$$\boldsymbol{2k_+M_e^2 = 2k_-D_e} \tag{10}$$

From equations (8), (9) and (10),

$$\frac{1}{\tau'^2} = 8k_+(2k_-D_e + k_-M_e) + k_-^2 = 8k_+k_-(2D_e + M_e) + k_-^2 = k_-^2 + 8k_+k_-M_{tot}$$

So, equation (7) can be rewritten as:

$$\boldsymbol{x = \frac{\frac{x(0)}{4k_+}\sqrt{k_-^2 + 8k_+k_-M_{tot}}}{\left(x(0) + \frac{1}{4k_+}\sqrt{k_-^2 + 8k_+k_-M_{tot}}\right) exp\left[\sqrt{k_-^2 + 8k_+k_-M_{tot}}t\right] - x(0)}} \tag{11}$$

In order to estimate the time constant of the BL-induced equilibrium shift, we next derived the time (τ) required for $x(t)$ to reduce to 1/e of its initial value.

Since $x(\tau)/x(0) = 1/e$, τ will be solved as follows:

$$\tau = \frac{1}{\sqrt{k_-^2 + 8k_+k_-M_{tot}}} ln\left(\frac{4k_+x(0)/\sqrt{k_-^2 + 8k_+k_-M_{tot}} + e}{4k_+x(0)/\sqrt{k_-^2 + 8k_+k_-M_{tot}} + 1}\right)$$

M_e and D_e can be described using k_+, k_- and M_{tot}, first by eliminating D_e from equations (8) and (10).

$$k_+ M_e^2 - k_- \left(\frac{M_{tot} - M_e}{2}\right) = 0$$

Then, since $M_e > 0$,

$$M_e = -\frac{k_-}{4k_+} + \frac{1}{4k_+}\sqrt{k_-^2 + 8k_+k_-M_{tot}}$$

From equation (8),

$$D_e = \frac{M_{tot}}{2} + \frac{k_-}{8k_+} - \frac{1}{8k_+}\sqrt{k_-^2 + 8k_+k_-M_{tot}}$$

Overall,

$$\boldsymbol{M *= M_e + \frac{\frac{x(0)}{2k_+}\sqrt{k_-^2 + 8k_+k_-M_{tot}}}{\left(x(0) + \frac{1}{4k_+}\sqrt{k_-^2 + 8k_+k_-M_{tot}}\right) exp\left[\sqrt{k_-^2 + 8k_+k_-M_{tot}}\, t\right] - x(0)}} \tag{12}$$

$$\boldsymbol{D *= D_e - \frac{\frac{x(0)}{4k_+}\sqrt{k_-^2 + 8k_+k_-M_{tot}}}{\left(x(0) + \frac{1}{4k_+}\sqrt{k_-^2 + 8k_+k_-M_{tot}}\right) exp\left[\sqrt{k_-^2 + 8k_+k_-M_{tot}}\, t\right] - x(0)}} \tag{13}$$

$$\boldsymbol{\tau = \frac{1}{\sqrt{k_-^2 + 8k_+k_-M_{tot}}} ln\left(\frac{4k_+x(0)/\sqrt{k_-^2 + 8k_+k_-M_{tot}} + e}{4k_+x(0)/\sqrt{k_-^2 + 8k_+k_-M_{tot}} + 1}\right)} \tag{14}$$

$$\boldsymbol{M_e = -\frac{k_-}{4k_+} + \frac{1}{4k_+}\sqrt{k_-^2 + 8k_+k_-M_{tot}}} \tag{15}$$

$$\boldsymbol{D_e = \frac{M_{tot}}{2} + \frac{k_-}{8k_+} - \frac{1}{8k_+}\sqrt{k_-^2 + 8k_+k_-M_{tot}}} \tag{16}$$

<u>Reference</u>

1. Akiyama, Y., Nakasone, Y., Nakatani, Y., Hisatomi, O. & Terazima, M. Time-Resolved Detection of Light-Induced Dimerization of Monomeric Aureochrome-1 and Change in Affinity for DNA. *J. Phys. Chem. B* 120, 7360–7370 (2016).
